# Recrystallization Behavior of MgAl Layered Double Hydroxide Films on Anodized Magnesium Alloys During Phytic Acid Loading

**DOI:** 10.3390/nano16201292

**Published:** 2026-10-12

**Authors:** Jiahao Wu, Wenjun Ci, Yuanfang Deng, Jiajia Yuan, Junlan Li, Xu Dai

**Affiliations:** 1 Basalt Fiber and Composite Key Laboratory of Sichuan Province, School of Modern Industry for Basalt Fiber Composite Materials, Sichuan University of Arts and Science, Dazhou 635000, China; 20250222@sasu.edu.cn (J.Y.); junlan0410@163.com (J.L.); 2 Dazhou Key Laboratory of Advanced Technology for Fiber Materials, School of Modern Industry for Basalt Fiber Composite Materials, Sichuan University of Arts and Science, Dazhou 635000, China; 3 School of Intelligent Manufacturing Engineering, Guangdong Polytechnic Normal University, Guangzhou 510665, China; ciwenjun0306@163.com; 4 Dazhou Market Supervision and Administration Bureau, Dazhou 635000, China; 13730798888@139.com; 5 Electric Power Research Institute CSG EHV Power Transmission Company, Guangzhou 510663, China; xudai1115@163.com

**Keywords:** anodic oxidation, corrosion inhibitor loading, corrosion resistance, layered double hydroxides, recrystallization

## Abstract

MgAl layered double hydroxides (LDHs) films were prepared via an in-situ growth method on anodized AZ31 magnesium alloy. Subsequently, phytic acid (PA) was loaded as a corrosion inhibitor through an anion-exchange process. The structural evolution of the LDHs nanosheets during the loading process was systematically investigated. The structure, morphology, and composition of the films were characterized using field-emission scanning electron microscopy, X-ray diffraction, energy-dispersive X-ray spectroscopy, Fourier transform infrared spectroscopy, and glow discharge optical emission spectroscopy. The corrosion behavior was evaluated using potentiodynamic polarization curves and electrochemical impedance spectroscopy. The results revealed that during the first four hours of ion exchange in the PA solution, the LDHs nanosheets underwent a fragmentation–recrystallization process. The incorporation of the corrosion inhibitor significantly enhanced both the stability of the LDHs nanosheets and the long-term corrosion protection of the substrate. Furthermore, a recrystallization mechanism for the LDHs nanosheets on the anodized AZ31 Mg alloy is proposed.

## 1. Introduction

Magnesium and its alloys are in high demand for lightweight structural applications, particularly in the electronics, transportation, and automotive industries. However, Mg alloys are prone to forming an oxidized-hydroxy-carbonate composite film upon exposure to the atmosphere. This native film fails to provide adequate passivation and protection for the Mg-based substrate, rendering Mg alloys more susceptible to corrosion than other metallic alloys and thereby limiting their widespread industrial application [1,2,3]. To overcome this issue, surface treatment technologies have become effective strategies for preventing the corrosion of magnesium alloys, such as by protecting the metal substrate through the use of anodic oxide films. Moreover, the protective performance of anodic oxide films can be further improved by adding additives such as Arsenazo-I to the electrolyte or by post-sealing treatment [4]. Notably, the combination of anodic oxide films and LDHs nanosheet sealing has led to significant advances in corrosion protection [5,6].

Layered double hydroxides (LDHs) are two-dimensional layered nanomaterials with a brucite-like structure [6,7,8]. Their general chemical formula is [M^2+^_1−x_M^3+^_x_ (OH)_2_]^x+^(A^n−^)_x/n_·mH_2_O, where M^2+^ and M^3+^ represent divalent and trivalent metal cations in the hydroxide host layers, respectively, and A^n−^ denotes an *n*-valent interlayer anion. The partial substitution of M^2+^ by M^3+^ renders the host layers positively charged [9,10]. Consequently, organic or inorganic anions can be intercalated between the layers, rendering the host–guest structure of the LDHs nanocontainers electrically neutral [7,11]. In recent years, researchers have loaded various corrosion inhibitor ions and other functional species into LDHs nanocontainers to achieve effective active corrosion inhibition [12]. Previous studies have demonstrated that loading phosphate [13], graphene [14,15], 1-hydroxyethylidene-1,1-diphosphonic acid [16], and other inhibitors [11,17] can effectively enhance the corrosion resistance of LDHs films. For instance, Sokol et al. [18] prepared Mg_2_Al-OH LDHs via an aqueous sol–gel method, followed by phosphate intercalation through successive anion-exchange reactions (OH^−^ → Cl^−^ → H_2_PO_4_^−^) assisted by high-power sonication, and elucidated the type and arrangement of the intercalated phosphate anions. Alibakhshi et al. [13] combined MgAl LDHs-PO_4_ nanoparticles with epoxy resin to form a protective coating on low-carbon steel. By employing an ion-exchange method to replace NO_3_^−^ with PO_4_^3−^, they proposed that the release of PO_4_^3−^ from the LDHs nanosheets is key to improving corrosion resistance. Chen et al. [19] utilized PA as an environmentally friendly corrosion inhibitor to modify LDHs films via adsorption and ion exchange, concluding that the resulting LDHs-PA film exhibits superior stability and long-lasting protection. Overall, numerous inorganic and organic eco-friendly corrosion inhibitor anions can be exchanged with interlayer anions in LDHs to enhance and prolong the protective properties of LDHs films. However, the behavior and evolution of LDHs nanosheets during this exchange process require further investigation to elucidate the effective corrosion protection mechanisms associated with inhibitor release from LDHs. LDHs films typically undergo anion-exchange reactions in a topotactic manner [20]. For example, Williams et al. [21] described ion exchange as a topotactic reaction based on the structural changes in the film before and after the reaction. However, the only structural change observed was related to the interlayer spacing of the LDHs, which depends on the spatial properties of the loaded anions. Kooli et al. [22] found that Mg-Cr-CO_3_^2−^ LDHs films exhibited significant weight loss during the ion exchange of vanadate in HCl solution at pH 4.5, with the formation of magnesium vanadate detected as a side reaction. They highlighted that ion exchange is a two-step process: LDHs first dissolve and then reprecipitate with foreign anions (the so-called dissolution–reprecipitation, or D-R, mechanism). Valente et al. [23] noted that the dissolution–recrystallization process of LDHs in alkaline solutions can occur upon the addition of other divalent metal cations, a phenomenon explained by Ostwald ripening theory. Ostwald ripening theory provides only a limited explanation for the growth process of LDHs [24]. In this study, we employed a variety of characterization techniques and data analyses to elucidate, in detail, the behavioral changes induced by the recrystallization of LDHs during the loading of corrosion inhibitors.

In this paper, Mg-Al-NO_3_ LDHs films were formed on the surface of anodized AZ31 magnesium alloy via an in-situ growth method. The samples were then immersed in an eco-friendly alkaline PA corrosion inhibitor solution for varying durations to obtain LDHs nanosheets at different evolutionary stages. Changes in properties such as morphology, structure, crystallinity, and composition of the nanosheets at various stages were investigated and discussed. Finally, a fragmentation–recrystallization mechanism for the LDHs nanosheets in the alkaline PA inhibitor solution was established.

## 2. Materials and Methods

### 2.1. Material

The AZ31 magnesium alloy substrate was supplied by the National Engineering Research Center for Magnesium Alloys, Chongqing, China. Its elemental composition (wt.%) is as follows: Al 3.0%, Zn 1.0%, Mn 0.5%, Ca 0.04%, Si 0.1%, Cu 0.05%, and balance Mg. AZ31 Mg alloy substrates of two different dimensions (10 mm × 10 mm × 5 mm and 20 mm × 20 mm × 5 mm) were used in this study. The substrates were polished with SiC paper up to 2000 grit on all surfaces. Subsequently, the substrates were ultrasonically cleaned in ethanol for 5 min and dried under an air stream. All chemical reagents were purchased from Sinopharm Chemical Reagent Co., Ltd., Shanghai, China.

### 2.2. Film Preparation

The anodic oxide film was formed for 30 min at an applied voltage of 20 V using a stabilized DC power supply (Gongyi Yuhua Instrument Co., Ltd., Gongyi, Henan, China). The electrolyte, containing 7.0 g/L sodium hydroxide (NaOH) and 3.5 g/L sodium aluminate (NaAlO_2_), was continuously stirred in the oxidation bath, with the electrolyte temperature maintained at 22 ± 3 °C by a water-cooling system. The electrolyte composition and anodizing conditions were selected based on well-established and experimentally validated protocols reported in previous studies on the fabrication of MgAl LDH films on anodized AZ31 magnesium alloy [5,25]. These conditions yield a uniform porous anodic oxide film that can serve as both an internal Al source and an anchoring layer for the subsequent in-situ growth of MgAl LDHs. The anodizing process was performed in a constant-voltage mode. The current density–time curve is shown in Figure S1. The initial current density was approximately 41.7 mA·cm^−2^, which rapidly decreased to approximately 1.8 mA·cm^−2^ within the first minute, and finally stabilized at approximately 2.8 mA·cm^−2^ after 30 min. During the formation of the anodic oxide film, a dynamic equilibrium exists between film formation and dissolution. Initially, the film grows rapidly, while the dissolution rate is negligible. As the film thickness gradually increases, the resistance increases accordingly, leading to a rapid decrease in current density. However, once the thickness reaches a certain value, the electric field at the bottom of the anodic pores is enhanced, which increases the dissolution rate and causes the current density to increase slowly. Eventually, the formation and dissolution rates tend to stabilize, the current density remains relatively constant, and the thickness of the porous outer layer continues to increase. The polished sample served as the anode, and a stainless-steel plate served as the cathode. The anodized samples were ultrasonically cleaned in ethanol and dried in air at room temperature.

**Figure 1:**
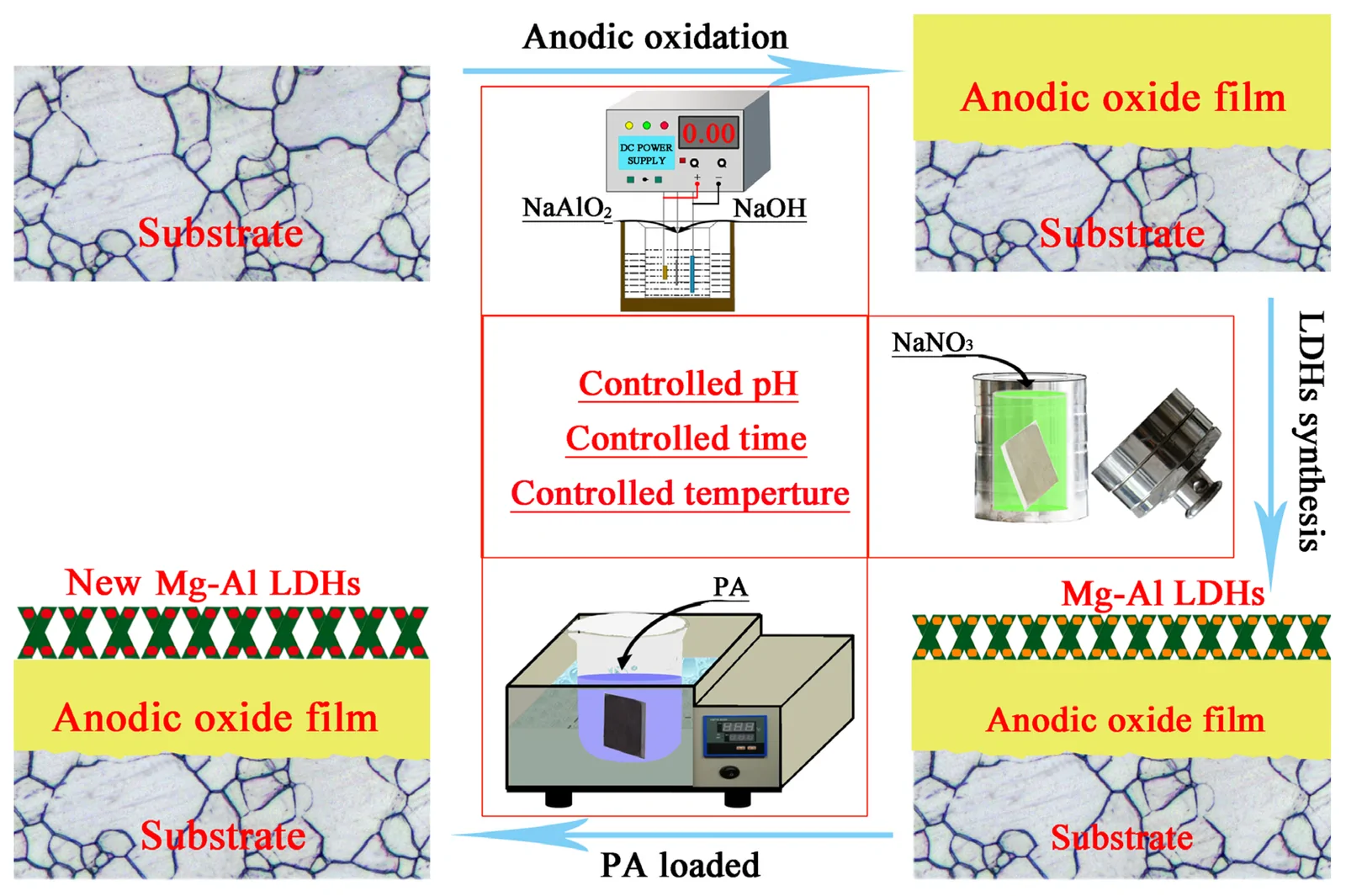
Schematic diagram of the preparation process of the PA-loaded MgAl LDHs film on the anodized AZ31 magnesium alloy. The process includes anodic oxidation, hydrothermal in-situ growth of MgAl–NO_3_ LDHs, and subsequent PA loading via anion exchange.

MgAl LDHs films on the anodized samples were produced via hydrothermal in-situ growth using a 0.1 M NaNO_3_ solution in a Teflon-lined autoclave at 125 °C for 12 h. The obtained samples were rinsed with deionized water and ethanol, then dried in air at room temperature. The as-prepared sample was designated as the “0 min” sample. The PA aqueous solution was prepared by diluting phytic acid (60 mL/L) with deionized water, and the pH was adjusted to 11 using NaOH. Each MgAl LDHs sample was immersed in 60 mL of the PA solution in a sealed glass vessel and kept at 80 °C in a thermostatic water bath. The samples were removed after immersion for 10 min, 30 min, 1 h, 2 h, and 4 h, respectively. After loading, the samples were gently rinsed with deionized water and ethanol and then dried with compressed air. The PA loading process was repeated three times for each condition to ensure reproducibility. The schematic diagram of PA loading onto the MgAl LDHs film is illustrated in Figure 1.

### 2.3. Characterization

The surface morphologies of the samples were examined using a field-emission scanning electron microscope (FE-SEM, JEOL JSM-7800F, Akishima, Tokyo, Japan). The elemental composition of the LDHs films was analyzed using energy-dispersive X-ray spectroscopy (EDS, INCA Energy 350, Oxford Instruments, Abingdon, UK). All samples were sputter-coated with gold. X-ray diffraction (XRD) analysis of the sample surfaces was conducted using a Rigaku D/Max 2500X X-ray diffractometer (Rigaku Corporation, Akishima, Tokyo, Japan) with Cu-Kα_1_ radiation (λ = 0.154 nm) over a 2θ range of 5° to 80° at a scan rate of 4°/min. A Nicolet iS5 FT-IR spectrometer (Thermo Fisher Scientific, Waltham, MA, USA) equipped with an attenuated total reflectance (ATR) accessory (scanning range: 400–4000 cm^−1^) was used for qualitative component analysis of the samples. Glow discharge optical emission spectroscopy (GDOES; GD-Profiler 2, Horiba Scientific, Kyoto, Japan) was employed for depth profile analysis of the different samples at a pressure of 700 Pa and a power of 40 W.

Electrochemical tests were performed using a Princeton Applied Research P4000A electrochemical workstation (AMETEK, Berwyn, PA, USA). A standard three-electrode cell was employed, consisting of a high-purity platinum sheet as the counter electrode, a saturated calomel electrode (SCE) as the reference electrode, and the sample as the working electrode. The test solution was a 3.5 wt.% NaCl aqueous solution, and the exposed area was 1 cm^2^. Once the open-circuit potential (OCP) stabilized, EIS measurements were conducted with an AC amplitude of 10 mV (vs. OCP) over a frequency range of 10^5^ to 10^−2^ Hz. Equivalent circuits were selected, and the EIS data were fitted using ZView software (version 3.5, Scribner Associates Inc., Southern Pines, NC, USA) to extract the circuit component values. Potentiodynamic polarization (PDP) curves were recorded at a scan rate of 1 mV/s within a voltage range of ±0.5 V (vs. OCP). Three parallel samples were tested to ensure reproducibility.

## 3. Results and Discussion

### 3.1. Structural and Composition of the Films

**Figure 2:**
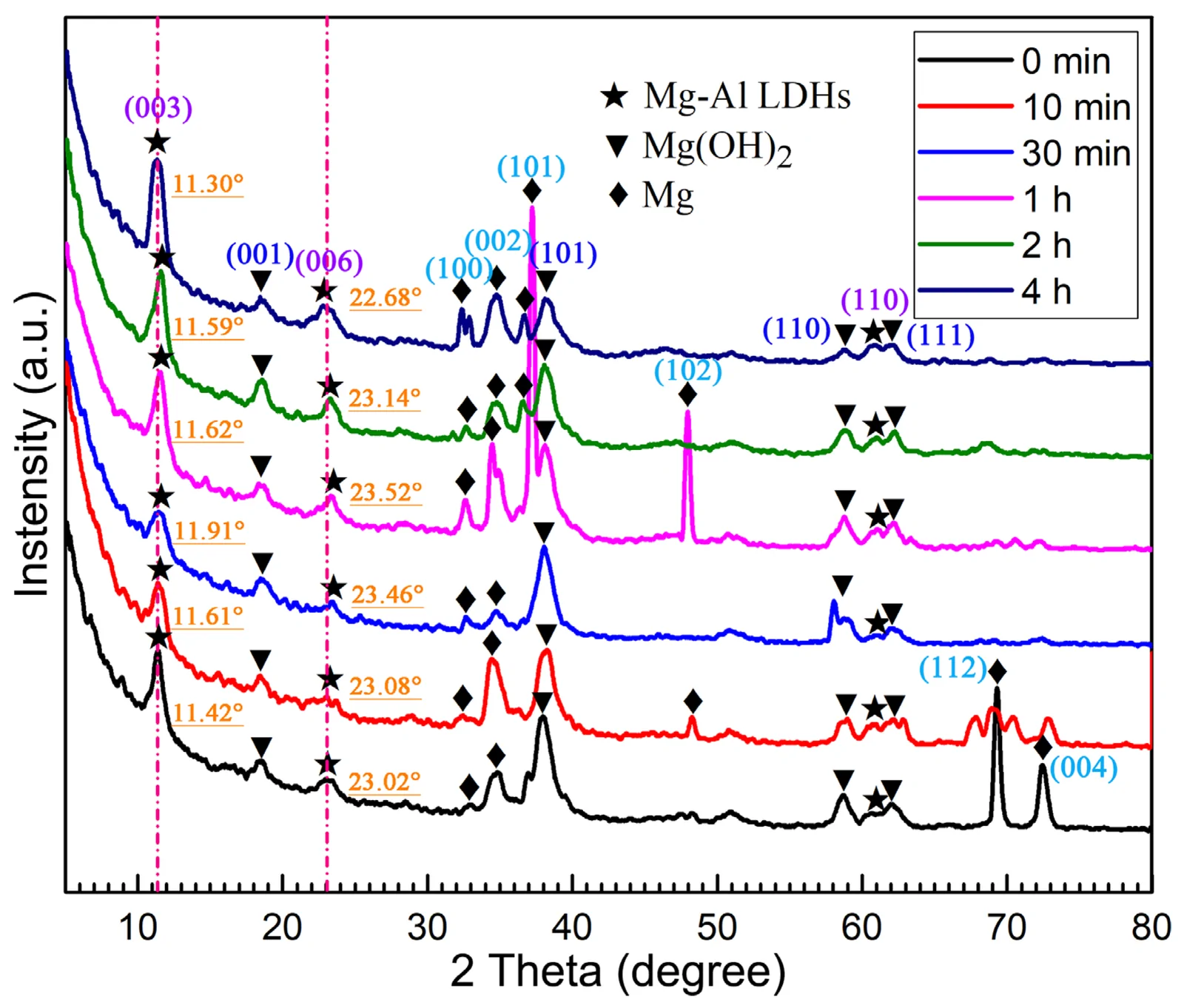
XRD patterns of the MgAl LDHs films loaded with PA for different durations (0 min, 10 min, 30 min, 1 h, 2 h, and 4 h). The characteristic diffraction peaks are labelled with their corresponding crystallographic planes: Mg–Al LDHs (003), (006) and (110); Mg(OH)_2_ (001), (101), (110), and (111); Mg (100), (002), (101), (102), (112), and (004).

The XRD patterns of the MgAl LDHs films loaded with PA for different durations (0 min, 10 min, 30 min, 1 h, 2 h, and 4 h) are presented in Figure 2. The diffraction peaks of the pristine MgAl LDHs (0 min) were observed at approximately 11.42°, 23.02° and 60.87°, corresponding to the (003), (006) and (110) crystallographic planes, respectively [25]. These reflections corresponded to a basal spacing of 7.74 Å [26]. In addition to the characteristic peaks of the LDHs, diffraction signals from the underlying layers were also detected due to the penetration depth of the X-rays. Specifically, the prominent peaks located at 32.91°, 34.60°, 36.82°, 47.95°, 68.82°, and 72.49° were assigned to the Mg alloy substrate, corresponding to the (100), (002), (101), (102), (112) and (004) planes, respectively. Furthermore, reflections at 18.60°, 38.02°, 58.67° and 62.13° were indexed to Mg(OH)_2_ for (001), (101), (110), and (111) planes, respectively. This was generated during the anodization pretreatment and the subsequent hydrothermal in-situ growth process. As shown in Figure 2, with the prolongation of immersion time, the characteristic diffraction peaks of the LDHs first shifted slightly to higher angles and then to lower angles, indicating that the basal spacing of the LDHs first decreased and then increased. The fitted (003) peak positions are summarized in Table S1. The (003) peak shifted from 11.42° (0 min) to 11.91° (30 min) and then to 11.30° (4 h), with fitting errors within ±0.02°. The observed shift is significantly larger than the fitting error, confirming that the trend is reliable and not caused by spectral noise. In particular, the diffraction angles of the (003) and (006) planes for the sample immersed for 4 h were both smaller than those of the unimmersed sample. Analogously, the intensity of the diffraction peaks exhibited a trend of decreasing first and then increasing during the immersion process, indicating that the crystallinity of the LDHs initially decreased and subsequently recovered [27]. LDHs with the highest crystallinity were obtained at the end of the immersion period.

**Figure 3:**
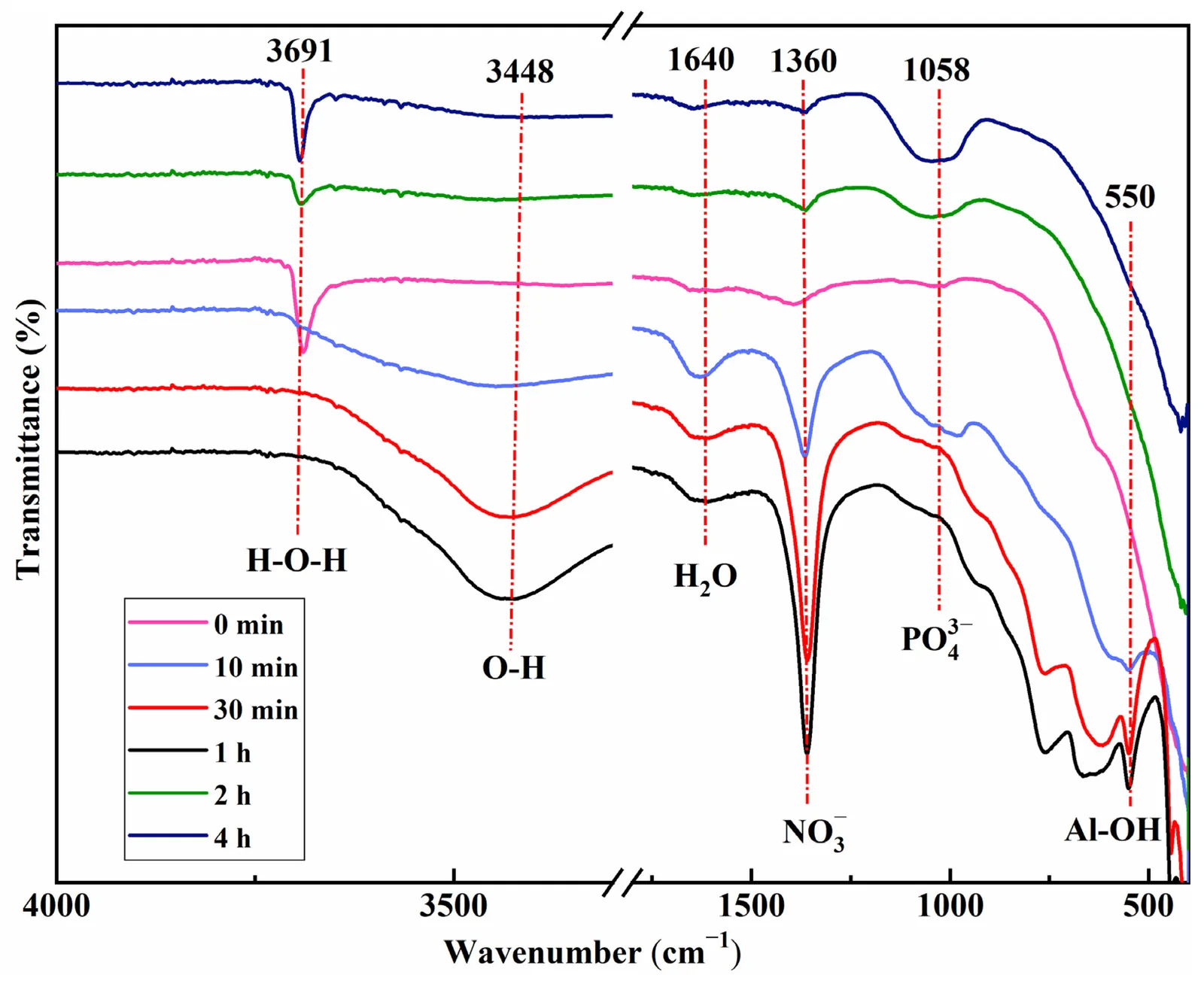
FT-IR spectra of the MgAl LDHs films loaded with PA for different durations (0 min, 10 min, 30 min, 1 h, 2 h, and 4 h). The breakpoints in the wavenumber range of 3300–1800 cm^−1^ are set to facilitate image display.

Figure 3 shows the FT-IR spectra of the MgAl LDHs films loaded with PA for different durations. Due to the absence of clear absorption peaks in the wavenumber range of 3300–1800 in the FT-IR curves of all samples, breakpoints were set during plotting to facilitate image display. The absorption peak at around 3691 cm^−1^ corresponds to the H–O–H stretching vibration. The absorption peak at 3448 cm^−1^ is attributed to the stretching mode of hydroxyl groups (O–H) from the host layers and interlayer water molecules [7,19]. The absorption peak at 1640 cm^−1^ is assigned to the bending vibration of water molecules (H_2_O) adsorbed on the film surface or intercalated within the LDHs nanosheets [28]. The absorption peak at around 1360 cm^−1^ corresponds to the stretching vibration of NO_3_^−^ anions, indicating that NO_3_^−^ anions were likely present in the interlayer of the LDHs nanosheets. With the increase in PA loading time, this absorption peak gradually attenuated, signifying that the NO_3_^−^ anions between the cationic layers of the LDHs nanosheets were gradually released during this process. Meanwhile, a new absorption peak emerged at around 1058 cm^−1^, which is related to the phytate anion (R-PO_4_^2−^) [13], indicating the interaction between the MgAl LDHs and PA [29]. The absorption peak at 550 cm^−1^ is associated with the Al–OH stretching [28]. Combined with the SEM and XRD analyses, the variation in the 1360 cm^−1^ absorption peak in the FT-IR spectra could be associated with the release of NO_3_^−^ during the dissolution of the MgAl LDHs nanosheets. The intensity transition of the 1058 cm^−1^ absorption peak was attributed to the anion exchange process capturing the high-affinity phytate anions.

**Figure 4:**
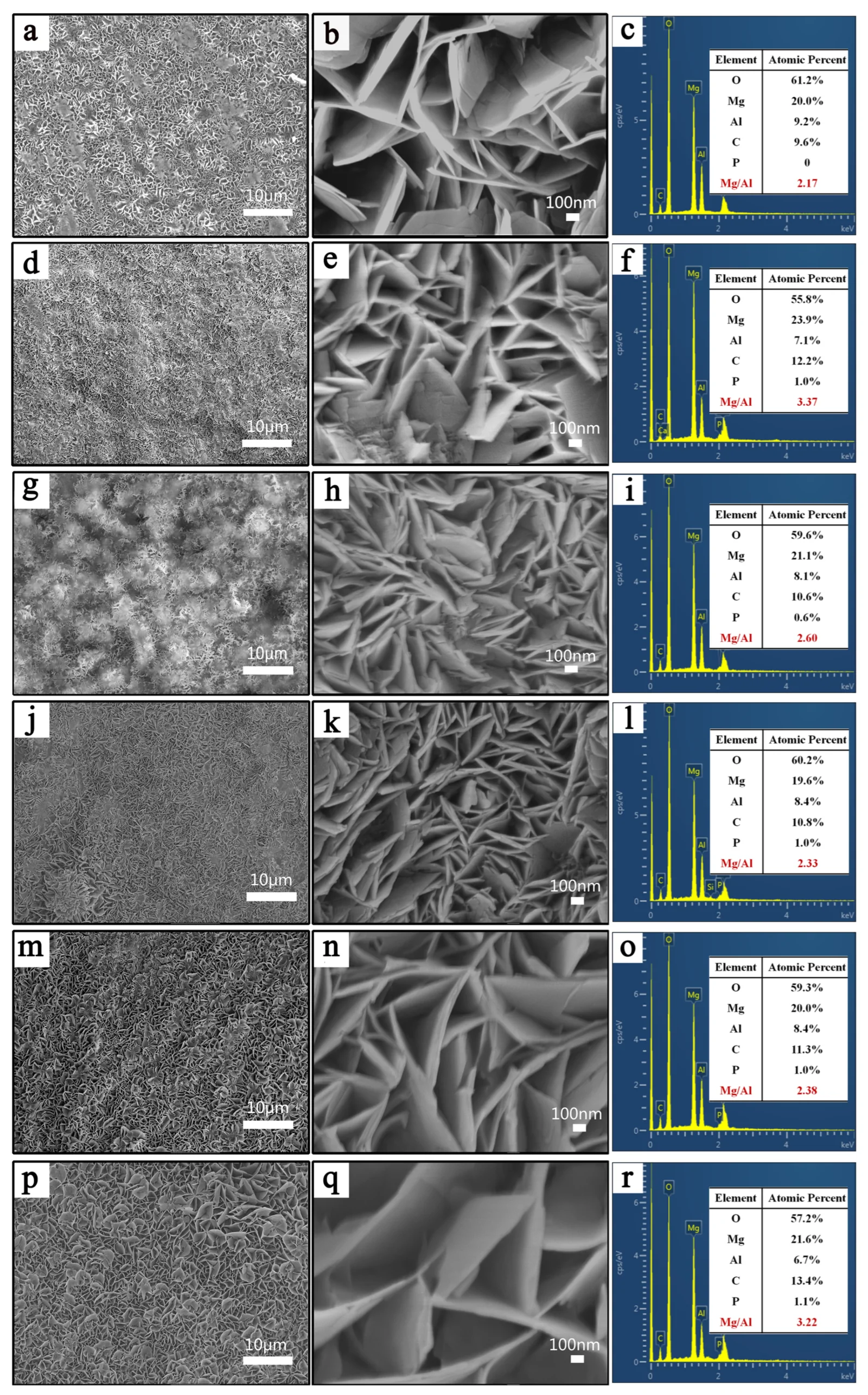
Surface SEM morphologies and corresponding EDS spectra of the MgAl LDHs films loaded with PA for different durations: (**a**–**c**) 0 min, (**d**–**f**) 10 min, (**g**–**i**) 30 min, (**j**–**l**) 1 h, (**m**–**o**) 2 h, and (**p**–**r**) 4 h. The left column shows SEM images, and the right column shows the corresponding EDS spectra.

The surface SEM morphologies and EDS spectra of the MgAl LDHs films loaded with PA for different durations are shown in Figure 4. The LDHs nanosheets exhibited a platelet morphology, which is the typical LDHs sheet-like structure with the size of the hexagonal plane (ab-faces) much larger than the thickness (c-axis) [30]. It was observed that the LDHs nanosheets (Figure 4a,b) were widely distributed on the substrate in a stacked sheet form with a mesh-like structure, and the density was relatively uniform, suggesting that the LDHs film could effectively protect the entire substrate. Additionally, it was found from Figure 4a that some of the LDHs nanosheets were covered by precipitates, which was related to the excess Mg(OH)_2_ in the growth solution. When the sample was immersed for 10 min, the nanoflower-like surface of the film diminished and became covered with more precipitates, as shown in Figure 4d. Furthermore, the LDHs nanosheets (Figure 4e) appeared slightly fractured and decomposed [13]. After immersion in PA for 30 min, the film surface became uneven, and pores appeared in certain locations (Figure 4g). The nanosheets in Figure 4h exhibited a small and densely stacked morphology, and the flower-like structure of the LDHs was partially disrupted, likely due to the further dissolution and fragmentation of the LDHs nanosheets. Upon immersion for 1 h, the film surface became flat, and compact, fine nanosheets were visible (Figure 4j). The high-magnification image (Figure 4k) revealed that the nanosheets were densely stacked, with numerous small-sized LDHs nanosheets appearing between the large-sized ones. After immersion for 2 h, Figure 4m showed that the flower-like nanosheets had regrown, and the precipitates on the film surface had decreased. The enlarged image revealed that the LDHs nanosheets possessed a complete structure and uniform size, similar to those in Figure 4b. Upon immersion for 4 h, the low-magnification images showed that the LDHs nanosheets had grown further, with distinct flower-like nanosheets, and some LDHs nanosheets had expanded outward and coarsened (Figure 4p). Thicker and more intact nanosheets were obtained compared to the unimmersed MgAl LDHs nanosheets (Figure 4q) [31]. Overall, it could be concluded that during immersion in the alkaline PA inhibitor solution, the LDHs nanosheets first dissolved, refined, and fragmented within the first hour, then regrew and coarsened from 1 and 4 h, a process that could be described as fragmentation–recrystallization growth [32].

Meanwhile, the strong signal of oxygen (O) was primarily related to the overall signal from MgO, Al_2_O_3_, Al(OH)_3_, Mg(OH)_2_, CO_3_^2−^, and PA. The higher carbon (C) content could be attributed to the carbonate and PA [33], as well as the residue of ethanol. During PA loading, the content of phosphorus (P) changed. The Mg/Al ratio was also inconsistent at different loading times, as shown in Figure 4c,f,i,l,o,r. These results demonstrated the compositional changes in the MgAl LDHs during PA loading.

**Figure 5:**
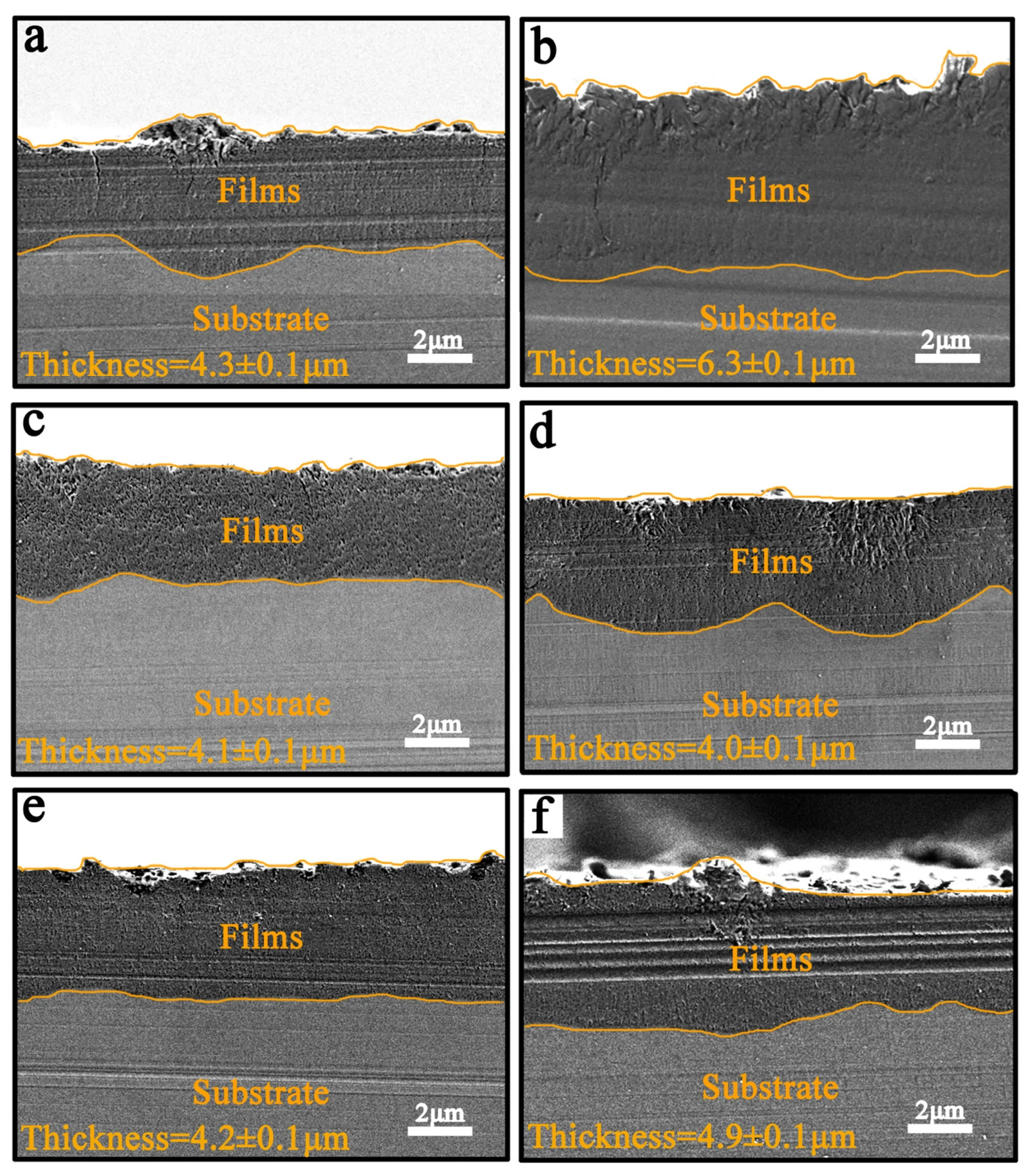
Cross-sectional SEM images of the MgAl LDHs/anodic oxide composite films before and after PA loading for different durations: (**a**) 0 min, (**b**) 10 min, (**c**) 30 min, (**d**) 1 h, (**e**) 2 h, and (**f**) 4 h. The average film thicknesses are approximately 4.3 ± 0.1, 6.3 ± 0.1, 4.1 ± 0.1, 4.0 ± 0.1, 4.2 ± 0.1, and 4.9 ± 0.1 μm, respectively.

Figure 5 presents the cross-sectional SEM images of the films before and after loading with PA for different durations. The structures of the films were compact and closely integrated with the substrate (Figure 5a–f). It was observed that the MgAl LDH nanosheets successfully penetrated the pores of the anodic oxide to form a composite film (Figure 5a), with an average thickness of approximately 4.3 ± 0.1 µm. However, the film thickness was relatively uneven, which was attributed to the localized concentration of current density during anodic oxidation. After PA loading for 10 min, the composite film became porous, and the overall thickness of the layer increased to 6.3 ± 0.1 µm (Figure 5b). This was likely due to the partial dissolution of the LDH nanosheets in the PA solution, accompanied by the release of ions and the subsequent generation of precipitates on the surface. After PA loading for 30 min, the thickness of the composite film decreased to 4.1 ± 0.1 µm (Figure 5c), which was related to the rapid dissolution of the LDH nanosheets via ion exchange in the PA solution. Figure 5d shows that the film became relatively uneven again after 1 h of immersion. This coating exhibited the smallest thickness among all the films, measuring approximately 4.0 ± 0.1 µm. Notably, large amounts of Mg^2+^ and Al^3+^ were released into the solution during this dissolution process, providing the ion sources for co-deposition to form new LDHs. It could be speculated that recrystallization began when the PA reacted with the original LDHs for 1 h, as also indicated by the surface SEM results discussed above. After PA loading for 2 h, the composite film became relatively uniform, and the thickness increased to 4.2 ± 0.1 µm (Figure 5e). This could be attributed to the co-deposition rate exceeding the dissolution rate [29]. After the sample was loaded for 4 h, the composite film became thicker than the original film (Figure 5a), reaching a thickness of 4.9 ± 0.1 µm (Figure 5f). This increase could be related to the formation of new LDH nanosheets with a more complete and stable structure [7,11].

**Figure 6:**
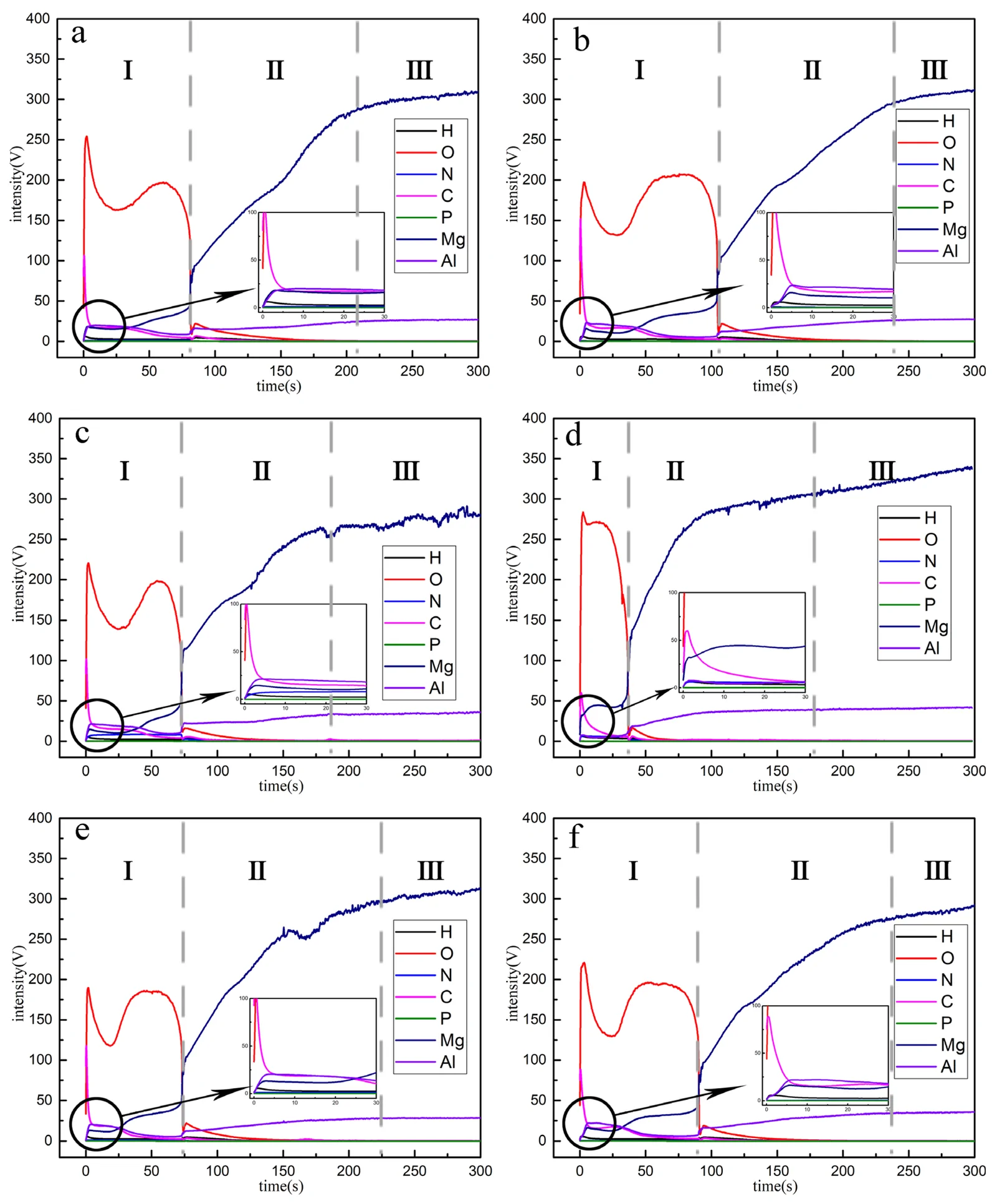
GDOES elemental depth profiles of the MgAl LDHs films loaded with PA for different durations: (**a**) 0 min, (**b**) 10 min, (**c**) 30 min, (**d**) 1 h, (**e**) 2 h, and (**f**) 4 h. Subareas I, II, and III correspond to the film layer, the film/substrate transition layer, and the metal substrate, respectively.

Figure 6 presents the GDOES elemental depth profiles of the MgAl LDHs films loaded with PA for different durations. There were roughly three regions in each of the coatings: (Subarea I) the film layer, (Subarea II) the film/substrate transition layer, and (Subarea III) the metal substrate. In the initial stage of sputtering (Figure 6a, Subarea I), the successful synthesis of the MgAl LDHs film was evidenced by the presence of O, Mg, Al, N, and H in the composite film, which was also confirmed by XRD and FT-IR. For the LDHs films loaded with PA (Figure 6b–f), Subarea I exhibited an overall trend of first narrowing and then widening, except for a minor abrupt change in Figure 6b. Moreover, the O element was present throughout the layer due to the loose and porous outer layer of the anodic oxide. LDHs nanosheets were grown in-situ exclusively on the surface of the anodized films. The Subarea I of the sample (10 min) became very wide, which might be because the LDHs nanosheets dissolved and released Mg^2+^ to react with phytate anion (R-PO_4_^2−^) in PA solution [13]. This phenomenon indicated that the elemental distribution and thickness of the LDHs/anodic oxide composite film had changed during PA inhibitor loading, which was also confirmed by SEM and FT-IR. On the one hand, as shown in Figure 6b,c (Subarea I), the contents of O and Al increased slightly, but the content of Mg decreased significantly, which may be due to the release of Mg^2+^ from partially dissolved LDHs nanosheets. In particular, the O and Mg contents (Figure 6d, Subarea I) reached their maximum values, but the scope became the narrowest. This was possibly because a large number of LDHs nanosheets in the outer layer of the composite film dissolved, exposing the inner anodic oxide film. These results demonstrated that the elemental composition on the sample surface changed significantly during PA inhibitor loading from 0 min to 1 h, which was related to the fragmentation and dissolution of the MgAl LDHs nanosheets. However, the elemental composition on the sample surface changed only slightly during PA inhibitor loading from 1 h to 4 h, which was related to anion exchange and recrystallization.

### 3.2. Corrosion Behavior of the Films

**Figure 7:**
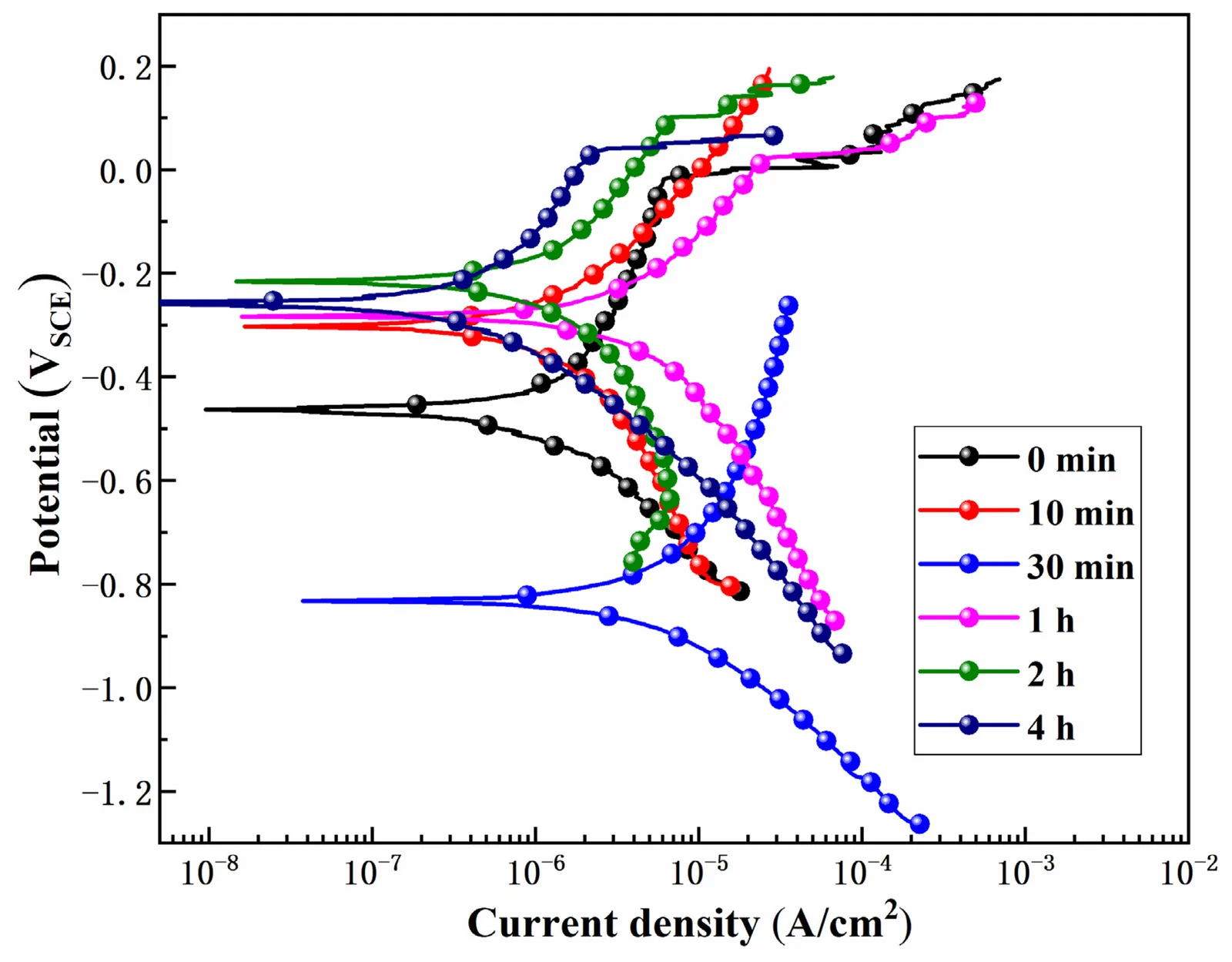
Potentiodynamic polarization curves of the MgAl LDHs films loaded with PA for different durations (0 min, 10 min, 30 min, 1 h, 2 h, and 4 h) measured in 3.5 wt.% NaCl solution.

The potentiodynamic polarization (PDP) curves measured in 3.5 wt.% NaCl for the MgAl LDHs films loaded with PA for different durations are presented in Figure 7. The measured and calculated parameters are presented in Table 1, where *E_corr_* and *i_corr_* are the corrosion potentials and the corrosion current densities, respectively [11,34]. The *E_corr_* and *i_corr_* values for the sample of parental MgAl LDHs film are −0.464 V_SCE_ and 0.514 μA/cm^2^, respectively. When the PA inhibitor treatment was performed for 10 min, the corrosion resistance of the LDHs film changed slightly, with *E_corr_* and *i_corr_* values of −0.319 V_SCE_ and 0.519 μA/cm^2^, respectively. This result indicated that ion deposition and transfer occurred on the sample immersed in the PA corrosion inhibitor for 10 min. After 30 min, a considerable change in *E_corr_* and *i_corr_* was recorded, shifting to −0.826 V_SCE_ and 1.810 μA/cm^2^, respectively. This indicated that the surface of the 30 min sample had been structurally damaged, its resistance to chloride ions was weakened, and the corrosion rate had increased during the corrosion process. For the sample immersed for 1 h, the corrosion resistance of the film improved slightly, leading to a significant positive shift in *E_corr_* to −0.287 V_SCE_ and a slight decrease in the *i_corr_* to 1.370 μA/cm^2^. For the 2 h sample, the densification of the surface film increased, resulting in a slight positive shift in the *E_corr_*, while *i_corr_* decreases significantly to 0.495 μA/cm^2^, with an *E_corr_* of −0.214 V_SCE_. After increasing the PA inhibitor treatment to 4 h, the corrosion resistance of the LDHs film was further improved, with *E_corr_* and *i_corr_* values of −0.255 V_SCE_ and 0.217 μA/cm^2^, respectively.

**Figure 8:**
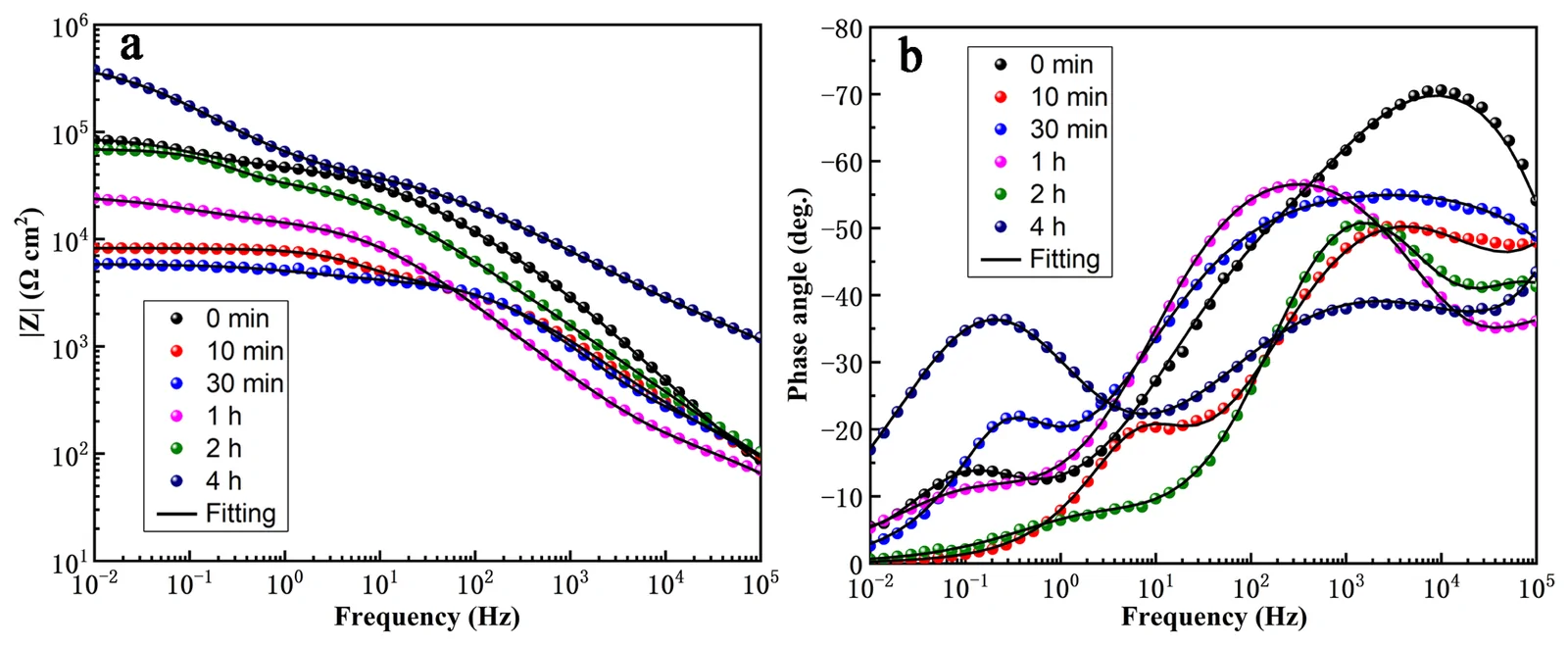
Bode plots of the MgAl LDHs films loaded with PA for different durations (0 min, 10 min, 30 min, 1 h, 2 h, and 4 h) measured in 3.5 wt.% NaCl solution: (**a**) impedance modulus |Z| vs. frequency and (**b**) phase angle vs. frequency.

The electrochemical characteristics of the MgAl LDHs films loaded with PA for different durations were analyzed through EIS testing. The surface potential was stabilized by soaking the sample for 30 min before the EIS test. Typically, the low-frequency impedance modulus (|*Z*| = 10^−2^ Hz) reflects the overall resistance of the films on the sample surface, thereby indicating the corrosion protection provided by the films to the substrate [34,35]. Figure 8a indicates that the overall resistance of the film first decreased and then increased during PA corrosion inhibitor loading. It is worth noting that when PA was loaded for 4 h, the maximum resistance value was observed, and the corrosion resistance was further improved compared to the 0-min sample. The single-layer or multi-layer structure of the film, the loose porous or tightly compact state, and the rates of charge transfer and diffusion all determine whether one, two, or more relaxation processes may occur in the EIS spectrum over a wide frequency range [11]. As shown in Figure 8b, there were two relaxation processes for the 0-min sample. First, the high-frequency region (10^4^ Hz) exhibited a large capacitance response corresponding to the residual porous outer layer of the anodic oxide and the loosely stacked LDHs nanosheet layer, followed by a smaller capacitance response in the low-frequency region (10^−1^ Hz) corresponding to the electrical double layer at the corrosion interface [36]. The phase angle–frequency diagram shows that the frequency corresponding to the relaxation process shifts, and the capacitance response increases and decreases during PA loading. Furthermore, to further discuss the corrosion mechanism, a physical model was used to simulate the respective responses. The fitting curves in Figure 8 were obtained using the equivalent circuits shown in Figure S2, and the detailed fitting parameters are summarized in Table S2. Two equivalent circuits were used: one for the 0 min, 10 min, 1 h, 2 h, and 4 h samples, and the other for the 30 min sample. In general, the *R_ct_* value determines the corrosion resistance of the sample. The *R_ct_* values of the 10 min and 30 min samples were much lower than that of the 0 min sample, with the 30 min sample showing the minimum *R_ct_* (3.8 × 10^3^ Ω·cm^2^), indicating the worst corrosion resistance. This was related to the fragmentation and dissolution of the MgAl LDHs nanosheets in the PA solution. With further PA loading, the *R_ct_* increased sequentially for the 1 h, 2 h, and 4 h samples. The 4 h sample exhibited the maximum *R_ct_* (4.2 × 10^5^ Ω·cm^2^), which exceeded that of the 0 min sample (4.6 × 10^4^ Ω·cm^2^), indicating the best corrosion resistance. These results are consistent with the potentiodynamic polarization results. The variations in the other parameters (*R_por_*, *R_inn_*, and *CPE* values) are also consistent with the fragmentation–recrystallization process and are detailed in Table S1.

**Figure 9:**
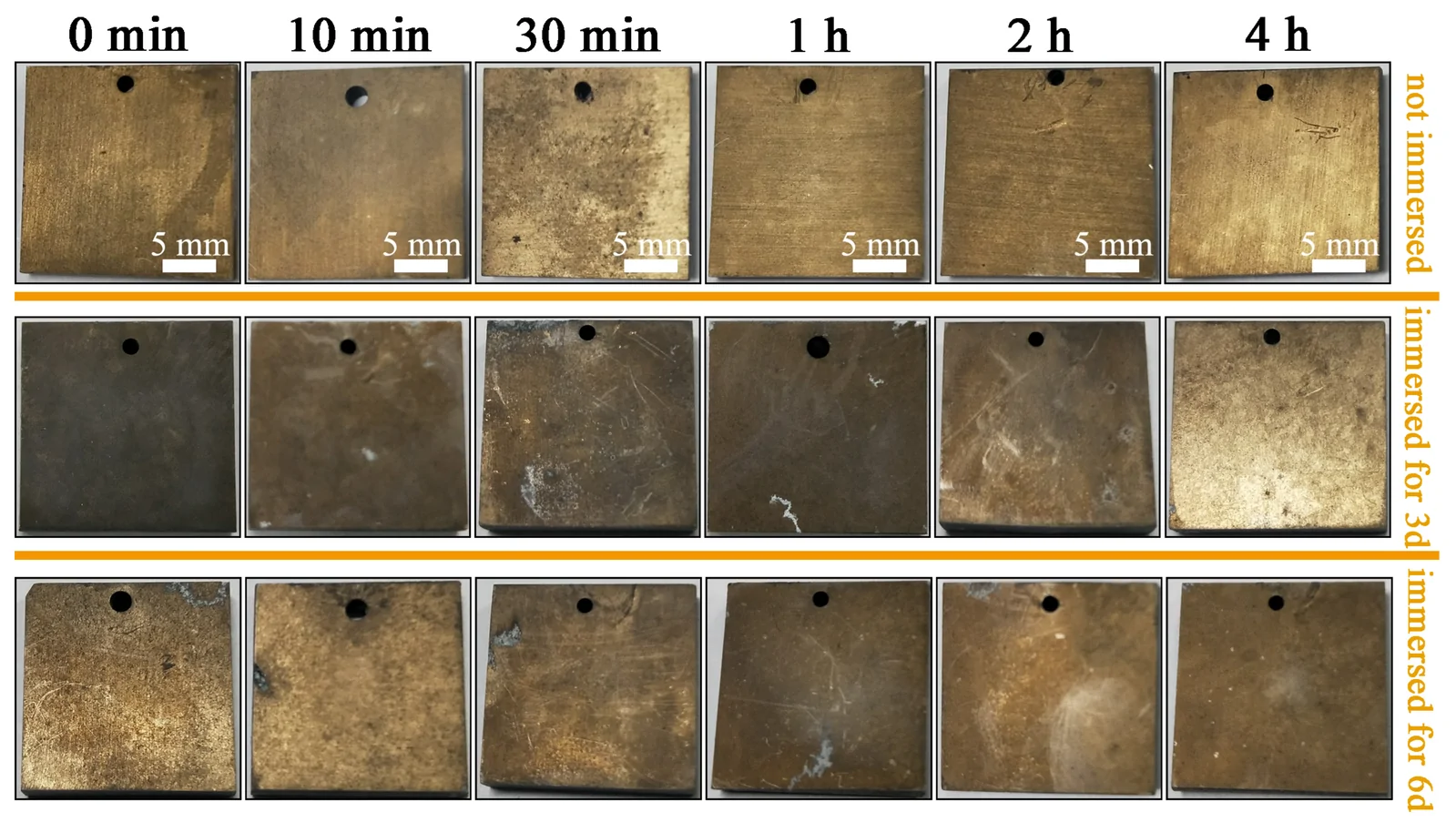
Macrographs of the MgAl LDHs films loaded with PA for different durations (0 min, 10 min, 30 min, 1 h, 2 h, and 4 h) after immersion in 3.5 wt.% NaCl solution for 0, 3 and 6 days.

Figure 9 presents the macrographs of the MgAl LDHs films loaded with PA for different durations after immersion in 3.5 wt.% NaCl solution for 0, 3 and 6 days. It could be seen from the initial samples (unimmersed) in Figure 9 that the surfaces of all the samples were relatively smooth and clean, except for a large number of pores on the surface of the 30 min sample. It could be speculated and confirmed by the EIS results that when the PA corrosion inhibitor was loaded for 30 min, the surface layer was dissolved and damaged, reducing a relaxation process, and the corrosion resistance was sharply attenuated. After immersion for 3 days, all the samples except the 4 h sample exhibited some amount of corrosion products on the surface, and the surfaces appeared dull. After immersion for 6 days, the surfaces of all the samples looked slightly dull. However, a relatively lower amount of corrosion products was observed for the 4 h sample.

Overall, the proposed PA-loaded MgAl LDHs film offers several advantages over the pristine MgAl–NO_3_ LDHs film. First, the electrochemical results demonstrate that PA loading significantly enhances the long-term corrosion protection of the anodized AZ31 magnesium alloy. After PA loading for 4 h, the *i*_corr_ decreased from 0.514 μA/cm^2^ (0 min) to 0.217 μA/cm^2^, and the *R*_ct_ increased from 4.6 × 10^4^ Ω cm^2^ (0 min) to 4.2 × 10^5^ Ω cm^2^, indicating a substantial improvement in corrosion resistance. The macrographs after immersion in 3.5 wt.% NaCl solution for 3 and 6 days also show that the 4 h sample had the least corrosion products. Second, phytic acid is an environmentally friendly and non-toxic corrosion inhibitor, which is beneficial for practical applications. Third, the fragmentation–recrystallization process converts the original MgAl–NO_3_ LDHs into a more stable phytate-intercalated MgAl LDHs structure, which not only improves the film stability but also provides a sustained release of phytate anions for active corrosion protection. Fourth, the combination of the anodic oxide film and the PA-loaded LDHs film provides dual protection: the anodic oxide film acts as a physical barrier and an internal Al source, while the LDHs film serves as a reservoir for the corrosion inhibitor. These advantages are supported by the XRD, FT-IR, SEM, GDOES, and electrochemical results presented in this work.

### 3.3. Recrystallization Mechanism of LDHs Nanosheets

Through the analysis of the above experimental results, it was found that both the structural composition and the corrosion resistance of the LDHs nanosheets changed regularly during PA ((CH_3_PO_4_)_6_) corrosion inhibitor loading. Combined with previous literature [37,38], the fragmentation and recrystallization mechanism of the LDHs nanosheets during the loading of the PA corrosion inhibitor was proposed.

Firstly, during the process of adjusting the pH of the PA solution to 11 ((CHPO_4_^2−^)_6_), the reaction that occurred was represented by the dissociation equilibrium of phytic acid, as follows:

(CH_3_PO_4_)_6_ + 12 OH^−^ → (CHPO_4_^2−^)_6_ + 12 H_2_O (1)

**Figure 10:**
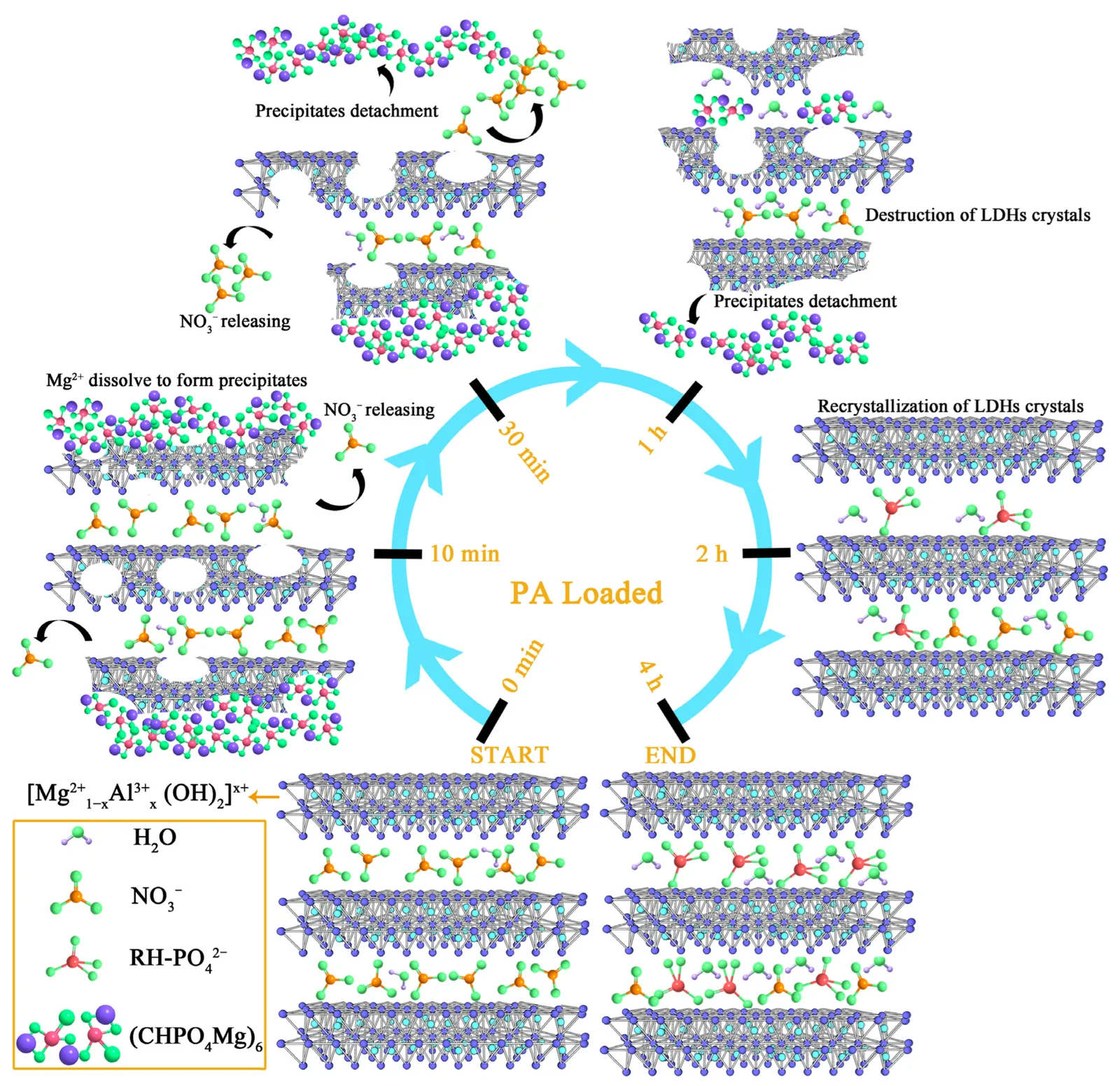
Schematic diagram of the fragmentation–recrystallization behavior of the MgAl LDHs nanosheets during PA loading. The fragmentation stage (0–1 h) and recrystallization stage (1–4 h) are marked.

Therefore, the phytate anions existed predominantly as (CHPO_4_^2−^)_6_ at the end of the pH adjustment of the solution [19]. Secondly, heating the adjusted solution to 80 °C not only increased the diffusion and exchange rates of ions in the solution but also increased the internal energy of the entire system, thereby increasing the Gibbs free energy of the solution. Then, after immersing the sample (0 min) with MgAl LDHs nanosheets into the solution, the composition and morphology of the film underwent a series of complex changes, as shown in Figure 10. In the early fragmentation stage of PA treatment, the main reactions of the LDHs nanosheets may occur as follows [39]:

[Mg_2_Al(OH)_6_]NO_3_·2H_2_O (s) ⇋ 2Mg^2+^(aq) + Al^3+^(aq) + 6OH^−^ + NO_3_^−^(aq) + 2H_2_O (2)

6Mg^2+^(aq) + (CHPO_4_^2−^)_6_(aq) → (CHPO_4_Mg)_6_(s)  K_sp1_ = 1.04 × 10^−24^ (3)

Mg^2+^(aq) + 2OH^−^ → Mg(OH)_2_(s)  K_sp2_ = 1.80 × 10^−11^ (4)

Al^3+^(aq) + PO_4_^3−^(aq) → Al(PO_4_)(s)  K_sp3_ = 6.30 × 10^−19^ (5)

Al^3+^(aq) + 3OH^−^ → Al(OH)_3_(s)  K_sp4_ = 1.30 × 10^−33^ (6)

**Figure 11:**
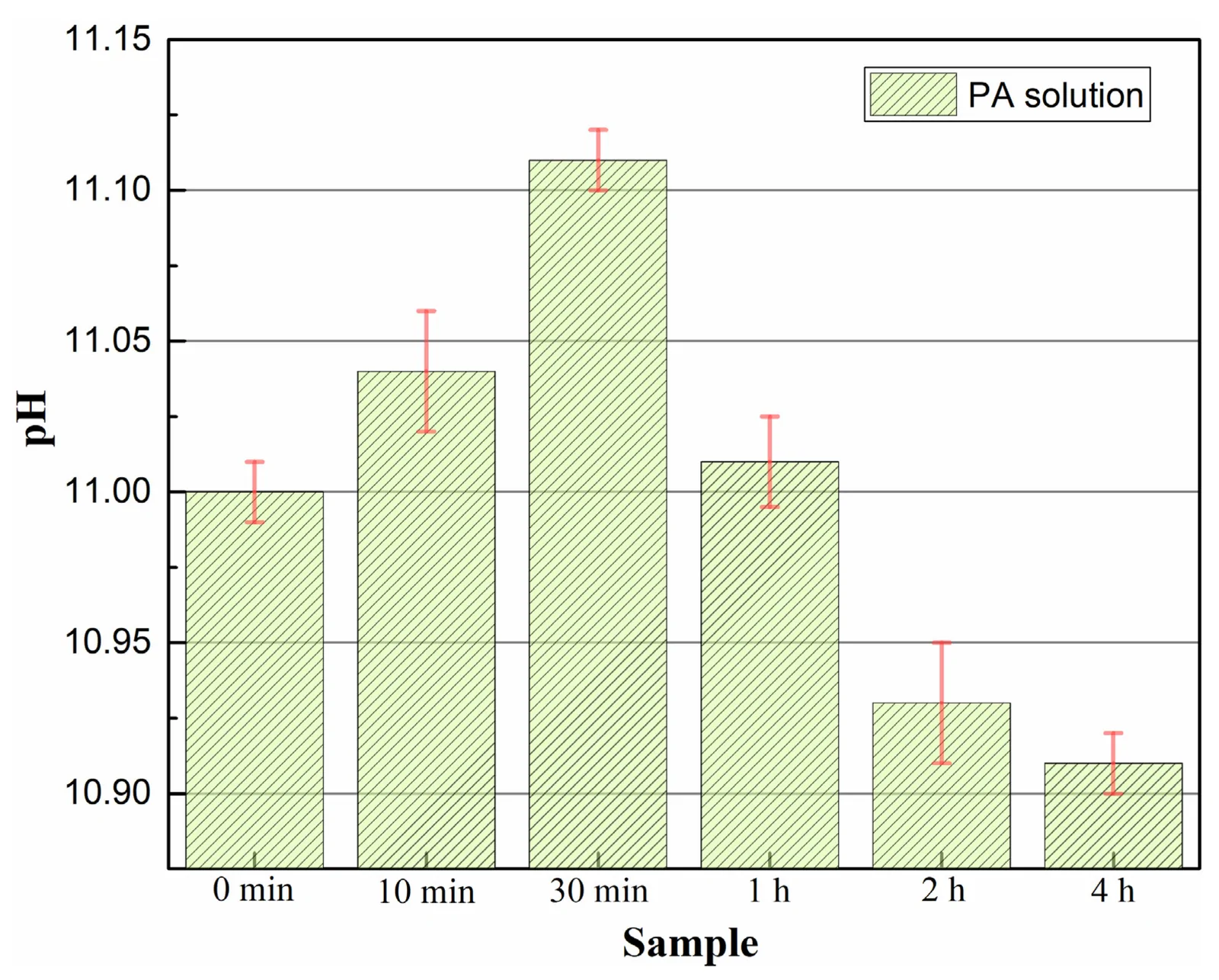
Changes in the pH value of the PA reaction solution during the PA loading process.

The concentration of PA (*c*_(*PA*)_) was 60 mL/L; therefore, the concentration of phosphate ($c_{\left({\mathrm{PO}}_{4}^{3-}\right)}$) was about 0.78 mol/L, and the concentration of hydroxide (*c*_(*OH*_^−^_)_) was 1.00 × 10^−3^ mol/L. The minimum Mg ion concentration ($c_{\min1\left({\mathrm{Mg}}^{2+}\right)}$) calculated by K_sp1_ of chemical Equation (3) was 1.20 × 10^−8^ mol/L, while the minimum Mg ion concentration ($c_{\min2\left({\mathrm{Mg}}^{2+}\right)}$) calculated by k_sp2_ of chemical Equation (4) was 1.80 × 10^−5^ mol/L. The minimum aluminum ion concentration ($c_{\min3\left({\mathrm{Al}}^{3+}\right)}$) calculated by K_sp3_ of the chemical Equation (5) was 8.08 × 10^−19^ mol/L, while the minimum aluminum ion concentration ($c_{\min4\left({\mathrm{Al}}^{3+}\right)}$) calculated by the k_sp4_ of the chemical Equation (6) was 1.30 × 10^−24^ mol/L [24]. Therefore, reactions (2) and (3) were the dominant processes within the first 10 min. Mg^2+^ detached from the nanosheets and combined with phytate anions at the contact interface to form (CHPO_4_Mg)_6_ precipitates covering the surface of the sample (10 min), as shown in Figure 10. In contrast, reaction (6) hardly proceeded under the alkaline condition, so Al^3+^ was not consumed by precipitation. The accumulation of Al^3+^ suppressed the forward dissolution of the MgAl LDHs; thus, reaction (2) proceeded slowly. A small amount of interlayer anions was released from the LDHs to maintain the charge neutrality of the LDHs nanosheets. As a result, the film became sparser and thicker due to the accumulation of (CHPO_4_Mg)_6_ precipitates [31]. During the treatment for 30 min (Figure 10), reaction (2) continued to occur, causing a large amount of LDHs nanosheets to fracture and dissolve, the (CHPO_4_Mg)_6_ precipitates fell off, and a large amount of interlayer anions were discharged [40]. This resulted in a decrease in the LDHs interlayer spacing, and the overall thickness of the film decreased sharply. The LDHs structure was even destroyed, and the film layer failed, which was reflected in the corrosion resistance tests. The XRD pattern (Figure 2) showed that the characteristic peak areas of (003) and (006) of the MgAl LDHs decreased continuously during PA treatment up to 1 h, indicating the existence of an LDHs fragmentation stage. In addition, as the PA treatment progressed to 1 h, the LDHs nanosheets entered the late recrystallization stage [41]. The kinetics of the observed recrystallization were similar to those observed by Turnbull [42]. The solid–liquid interface was in a completely wetted state at a constant temperature. When the Gibbs free energy of the solution reached the potential barrier, heterogeneous nucleation occurred on the original solid phase interface. As shown in Figure 10, the LDHs structure of the 1-h sample recrystallized to form some new LDHs nanosheets. Meanwhile, the (CHPO_4_Mg)_6_ precipitates reached saturation. Figure 11 presents the pH changes in the reaction solution during PA corrosion inhibitor treatment. In the early fragmentation stage, the pH value of the reaction solution increased continuously, which indicated that reaction (2) proceeded in the forward direction. This further proved that the LDHs nanosheets were dissolved and fragmented at this stage [32]. However, in the late recrystallization stage, the pH value of the reaction solution decreased continuously, which indicated that reaction (2) did not proceed or proceeded in the reverse direction. Meanwhile, considering the solution environment and the electronegativity of the anions, the recrystallization stage proceeded according to reaction (7), in which phytate anions were incorporated into the interlayer galleries of the newly formed LDHs. It should be noted that nitrate ions were the original interlayer anions of the as-prepared MgAl LDHs and maintained the charge balance of the LDHs. During PA loading, phytate anions partially replaced the interlayer nitrate ions through anion exchange, and the released nitrate ions entered the PA solution. The release of nitrate ions mainly occurred during the dissolution/fragmentation stage, whereas reaction (7) describes the recrystallization of new LDHs with phytate anions as the interlayer species.

24Mg^2+^(aq) + 12Al^3+^(aq) + 72OH^−^ + (CHPO_4_^2−^)_6_(aq) + mH_2_O → [Mg_2_Al(OH)_6_]_12_(CHPO_4_)_6_·mH_2_O(s) (7)

As the PA treatment progressed to 2 h (Figure 10), reaction (7) continued to occur, causing the pH value to decrease. As a result, most of the LDHs nanosheets regrew to restore the original structure, and the LDHs nanosheets became thicker [13]. After treatment for 4 h (Figure 10), the LDHs interlayer spacing increased slightly, as did the thickness of the LDHs nanosheets [24]. This could be explained by the Ostwald ripening phenomenon [41], where large crystals absorbed the mass of small crystals for coarsening growth, driven by the reduction in specific surface energy. The cross-sectional SEM images in Figure 5 provide experimental support for the schematic model in Figure 10. The film thickness first increased to 6.3 ± 0.1 μm at 10 min due to the formation of (CHPO_4_Mg)_6_ precipitates, then decreased to 4.0 ± 0.1 μm at 1 h due to the dissolution and fragmentation of the LDHs nanosheets, and finally increased to 4.9 ± 0.1 μm at 4 h due to recrystallization. These thickness changes are consistent with the fragmentation–recrystallization process illustrated in Figure 10. Therefore, the LDHs nanosheets underwent fragmentation and recrystallization during PA treatment. The SEM and XRD results showed that in the fragmentation stage of PA treatment (0–1 h), the size of the LDHs nanosheets gradually decreased, and the crystallinity of the LDHs nanosheets decreased. However, in the recrystallization stage (1–4 h), new small LDHs nanosheets were produced and gradually grew, and the crystallinity increased. In particular, the LDHs nanosheet structure of the 4-h sample was more stable, which was related to the formation of new MgAl LDHs via recrystallization replacing the original MgAl LDHs, according to the composition analysis results.

## 4. Conclusions

In this study, MgAl LDHs films were prepared on anodized AZ31 magnesium alloy, and phytic acid (PA) was loaded as a corrosion inhibitor via anion exchange. The results demonstrate that PA loading induces a fragmentation–recrystallization process of the MgAl LDHs nanosheets, converting the initial MgAl–NO_3_ LDHs into a more stable phytate-intercalated MgAl LDHs film and enhancing the long-term corrosion protection of the anodized Mg alloy. The key findings summarized are as follows:

(1) **Structural and morphological evolution.** SEM and XRD results showed that the LDHs nanosheets underwent fragmentation and recrystallization during PA loading. In the fragmentation stage (0–1 h), the size of the LDHs nanosheets gradually decreased, and the crystallinity and basal spacing decreased. In the recrystallization stage (1–4 h), new small LDHs nanosheets formed and gradually grew, and the crystallinity and basal spacing recovered and increased. The 4 h sample exhibited a more stable LDHs nanosheet structure, which is attributed to the formation of new phytate-intercalated MgAl LDHs via recrystallization, replacing the original MgAl–NO_3_ LDHs.

(2) **Corrosion resistance evolution.** Electrochemical results showed that the corrosion resistance of the MgAl–NO_3_ LDHs film first decreased, then increased with prolonged PA loading. The 10 min sample exhibited weakened corrosion resistance, and the 30 min sample exhibited the worst corrosion resistance, which was related to the failure of the LDHs film caused by the fragmentation of the LDHs nanosheets. Subsequently, the corrosion resistance was enhanced, and the 4 h sample exhibited the best corrosion resistance. This variation is consistent with the fragmentation–recrystallization process. The successful loading of PA improved the stability and long-term corrosion resistance of the LDHs film.

(3) **Fragmentation–recrystallization mechanism.** The mechanism is proposed as follows: (i) Mg^2+^ in the MgAl LDHs detached from the host layer structure and formed (CHPO_4_Mg)_6_ precipitates in the PA solution. The electroneutral state of the LDHs was disrupted, and NO_3_^−^ was released, causing further structural damage. This was reflected macroscopically in the dissolution and fragmentation of the LDHs nanosheets. (ii) After the (CHPO_4_Mg)_6_ precipitates reached saturation, at a constant temperature of 80 °C, new LDHs nanosheets began to nucleate heterogeneously on the residual Al(OH)_3_. The LDHs nanosheets adsorbed (CHPO_4_^2−^)_6_, which occupied the interlayer anion sites, and further coarsened to form a more stable MgAl LDHs structure.

**Outlook.** In future work, the PA loading time will be further optimized to maximize the long-term corrosion protection of the LDHs film. The self-healing ability of the PA-loaded LDHs film under prolonged immersion and mechanical damage will be systematically evaluated. In addition, this strategy will be extended to other magnesium alloys and complex-shaped components, and the industrial scalability of the combined anodizing/LDHs/PA process will be explored.

## Figures and Tables

**Table 1:** Parameters related to the polarization curves in Figure 7 for the MgAl LDHs films loaded with PA for different time.

Samples	*E_corr_* (V_SCE_)	*i_corr_* (µA/cm^2^)
0 min	−0.464	0.514
10 min	−0.319	0.519
30 min	−0.826	1.810
1 h	−0.287	1.370
2 h	−0.214	0.495
4 h	−0.255	0.217

## Data Availability

The original contributions presented in this study are included in the article/Supplementary Materials. Further inquiries can be directed to the corresponding author.

## Supplementary Materials

The following supporting information can be downloaded at: https://www.mdpi.com/article/10.3390/nano16201292/s1, Figure S1: Current density–time curve during anodizing of AZ31 magnesium alloy at a constant voltage of 20 V for 30 min in an electrolyte containing 7.0 g/L NaOH and 3.5 g/L NaAlO_2_; Figure S2: Equivalent circuits used to fit the EIS data; Table S1: Fitted (003) peak positions of the MgAl LDHs films loaded with PA for different durations; Table S2: Fitting results for EIS data of the MgAl LDHs films loaded with PA for different durations.
