# Supplementary material for: Recrystallization Behavior of MgAl Layered Double Hydroxide Films on Anodized Magnesium Alloys During Phytic Acid Loading
Source: Nanomaterials (Basel). 2026 Oct 12;16(20):1292. doi: 10.3390/nano16201292 (PMC13707670; doi:10.3390/nano16201292)
Supplement: Supplementary file 1 [file nanomaterials-16-01292-s001.zip › nanomaterials-4561480-supplementary.pdf]

nanomaterials

MDPI

Supplementary Material

Figure S1

Figure S1. Current density–time curve during anodizing of AZ31 magnesium alloy at a constant voltage of 20 V for 30 min in an electrolyte containing 7.0 g/L NaOH and 3.5 g/L $NaAlO_2$. The exposed area of the sample was 12 cm^2. The initial current density was approximately 41.7 $mA \cdot cm^{-2}$, which rapidly decreased to approximately 1.8 $mA \cdot cm^{-2}$ within the first minute and finally stabilized at approximately 2.8 $mA \cdot cm^{-2}$ after 30 min.

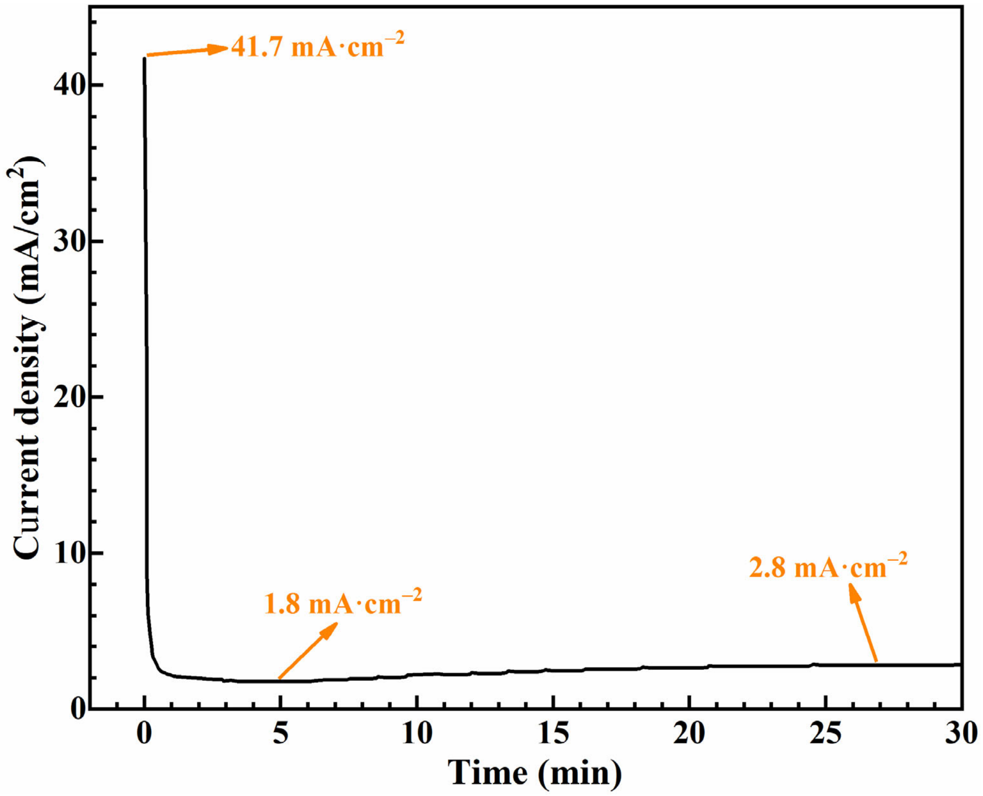

Figure S2

Figure S2. Equivalent circuits used to fit the EIS data: (a) 0 min, 10 min, 1 h, 2 h, and 4 h samples; (b) 30 min sample. R_{sol}, R_{por}, R_{inn}, and R_{ct} represent the resistance of the electrolyte, the loose outer layer, the dense inner layer, and the charge transfer, respectively. CPE_{por}, CPE_{inn}, and CPE_{dl} represent the constant phase elements corresponding to the loose outer layer, the dense inner layer, and the electrical double layer, respectively. The CPE impedance is defined as $Z_{CPE}=Q(j\omega)^{-n}$, where $0<n\leq1$.

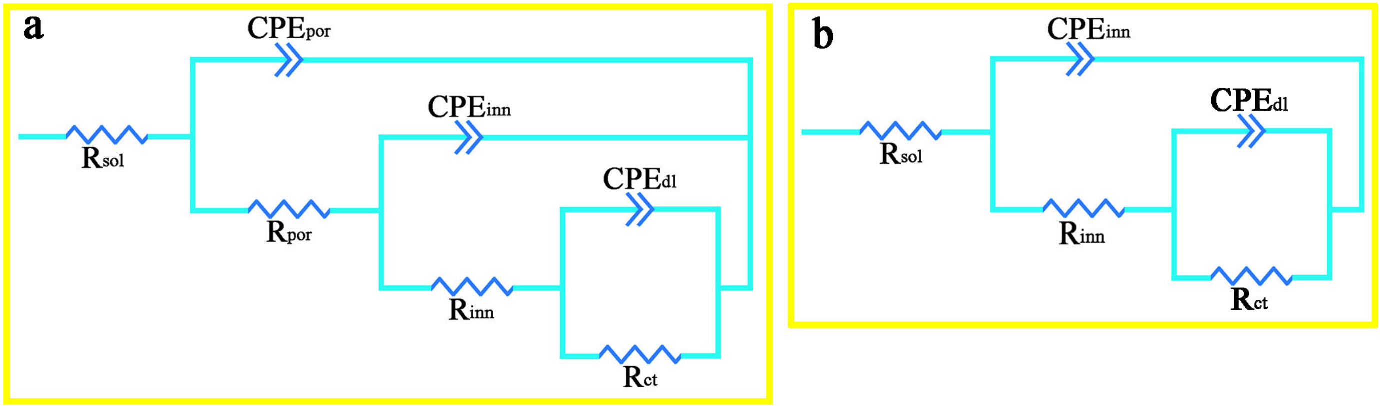

Table S1

Table S1. Fitted (003) peak positions of the MgAl LDHs films loaded with PA for different durations. The peak positions were determined by fitting the (003) reflection using Jade software. The fitting errors are within ±0.02°. This table supports the description of the peak shift in Fig. 2 and Section 3.1 of the main text.

Samples	(003) peak position (2θ, °)	Error (±°)
0 min	11.42	0.01
10 min	11.61	0.02
30 min	11.91	0.02
1 h	11.62	0.02
2 h	11.59	0.01
4 h	11.30	0.02

Note: The (003) reflection is the strongest and most characteristic basal reflection of the MgAl LDHs. The fitted peak positions show that the (003) peak first shifts to higher angles (from 11.42° at 0 min to 11.91° at 30 min) and then to lower angles (to 11.30° at 4 h) with increasing PA loading time. The observed shift is significantly larger than the fitting error, confirming that the trend is reliable and not caused by spectral noise.

Table S2

Table S2. Fitting results for EIS data of the MgAl LDHs films loaded with PA for different durations (corresponding to Fig. 8 in the main text). The EIS data were fitted using the equivalent circuits shown in Fig. S2. R_{sol}, R_{por}, R_{inn}, and R_{ct} represent the resistance of the electrolyte, the loose outer layer, the dense inner layer, and the charge transfer, respectively. CPE_{por}, CPE_{inn}, and CPE_{dl} represent the constant phase elements corresponding to the loose outer layer, the dense inner layer, and the electrical double layer, respectively. The CPE impedance is defined as $Z_{CPE}=Q(j\omega)^{-n}$, where $0<n\leq1$.

Samples	R_{por} (Ω cm²)	C_{por} (S sⁿ cm⁻²)	n_{por}	R_{inn} (Ω cm²)	C_{inn} (S sⁿ cm⁻²)	n_{inn}	R_{ct} (Ω cm²)	C_{dl} (S sⁿ cm⁻²)	n_{dl}	χ^2
0 min	6.6×10^3	1.7×10^{-7}	0.9	3.6×10^4	1.7×10^{-6}	0.6	4.6×10^4	4.9×10^{-5}	0.8	3.2×10^{-4}
10 min	1.3×10^2	1.6×10^{-7}	0.8	3.6×10^3	2.2×10^{-6}	0.7	4.5×10^3	1.2×10^{-5}	0.9	1.7×10^{-4}
30 min	---	---	---	3.1×10^4	2.5×10^{-6}	0.8	3.8×10^3	2.5×10^{-5}	0.8	6.9×10^{-4}
1 h	1.3×10^2	1.2×10^{-6}	0.7	1.1×10^4	2.8×10^{-6}	0.7	1.5×10^4	1.7×10^{-4}	0.7	2.9×10^{-4}
2 h	3.1×10^2	5.1×10^{-7}	0.7	1.8×10^3	6.5×10^{-7}	0.8	3.9×10^4	1.4×10^{-4}	0.6	3.9×10^{-3}
4 h	9.3×10^2	5.8×10^{-7}	0.9	5.0×10^4	1.2×10^{-6}	0.5	4.2×10^5	6.2×10^{-6}	0.7	5.6×10^{-4}

Note: For the 30 min sample, the EIS data were fitted with the equivalent circuit shown in Fig. S2b, in which R_{por} *and* CPE_{por}, are absent. The symbol "---" indicates that the corresponding parameter is not applicable or not available. The χ^2 values were negligible, indicating good fitting quality.
